# Regulation of A1 by OX40 Contributes to CD8^+^ T Cell Survival and Anti-Tumor Activity

**DOI:** 10.1371/journal.pone.0070635

**Published:** 2013-08-01

**Authors:** Fengyang Lei, Jianyong Song, Rizwanul Haque, Mohammad Haque, Xiaofang Xiong, Deyu Fang, Michael Croft, Jianxun Song

**Affiliations:** 1 Department of Microbiology and Immunology, The Pennsylvania State University College of Medicine, Hershey, Pennsylvania, United States of America; 2 Center of Irradiation, The Third Military Medical University, Chongqing, China; 3 Department of Pathology, Northwestern University Feinberg School of Medicine, Chicago, Illinois, United States of America; 4 Division of Immune Regulation, La Jolla Institute for Allergy and Immunology, La Jolla, California, United States of America; Karmanos Cancer Institute, United States of America

## Abstract

The TNFR family member OX40 (CD134) is critical for optimal clonal expansion and survival of T cells. However, the intracellular targets of OX40 in CD8 T cells are not fully understood. Here we show that A1, a Bcl-2 family protein, is regulated by OX40 in effector CD8 T cells. In contrast to wild-type T cells, OX40-deficient CD8 T cells failed to maintain A1 expression driven by antigen. Conversely, enforced OX40 stimulation promoted A1 expression. In both situations, the expression of A1 directly correlated with CD8 T cell survival. In addition, exogenous expression of A1 in OX40-deficient CD8 T cells reversed their survival defect in vitro and in vivo. Moreover, forced expression of A1 in CD8 T cells from OX40-deficient mice restored the ability of these T cells to suppress tumor growth in a murine model. These results indicate that OX40 signals regulate CD8 T cell survival at least in part through maintaining expression of the anti-apoptotic molecule A1, and provide new insight into the mechanism by which OX40 may impact anti-tumor immunity.

## Introduction

Costimulatory signals perform an important function in modulating adaptive and regulatory immunity. OX40 (CD134), a tumor necrosis factor receptor (TNFR) family member that is expressed by activated T lymphocytes, plays a critical role in maximizing proliferation, cytokine production, survival, and memory development of T cells [1]. Targeting OX40 in positive or negative ways with agonist or antagonist reagents, respectively, has shown promise for therapeutic intervention in cancer and infectious disease, as well as transplantation and autoimmunity.

While much of the initial data on OX40 related to control of CD4 T cells, many studies have now shown that OX40 is also important in promoting expansion and accumulation of effector and memory CD8 T cells [2]–[6]. In mouse studies of infectious disease, antigen specific CD8 T cell responses were compromised in the absence of OX40 after infection with influenza virus, cytomegalovirus, vaccinia virus, Listeria monocytogenes (Lm), or lymphocytic choriomeningitis virus (LCMV) [7]–[11]. Systemic injection of an agonist antibody to OX40 has also strongly enhanced the development of effector or memory CD8 T cells in basic systems [12], after virus infection [13], [14], and in models of tumor immunity [3], [15]–[19]. However, the intracellular targets of OX40 that regulate CD8 T cells have not been defined.

We have previously shown in CD4 T cells that OX40 sustained PKB (Akt) or IKKβ signaling leading to upregulation of several Bcl-2 family members (*e.g.*, Bcl-xL, Bcl-2) that controlled T cell longevity [20], [21]. Therefore, we investigated whether OX40 signals also targeted Bcl-2 family members to regulate CD8 T cell survival. The Bcl-2 prosurvival homolog A1 (mouse, 172 aa protein)/Bfl-1 (human, 175 aa protein) is mainly expressed in the hematopoietic system [22]. Overexpression of A1 in lymphoma cell lines significantly inhibited apoptosis induced by various stimuli such as TNF, FAS, TRAIL, and staurosporine (STS) [23]–[25]. In contrast, A1 knockdown B lymphoma cells were sensitive to apoptosis induced by CD20 cross-linking and DNA-damaging agents [26]. Furthermore, transgenic expression of A1 in the lymphoid compartment of mice improved B and T cell survival [27], [28]. Previous studies therefore suggest that A1 has a cytoprotective function, which may be essential for lymphocyte activation as well as cell survival. Using *in vitro* and *in vivo* approaches, as well as a tumor model, the studies presented here have identified and characterized A1 as an important target of OX40 signals to regulate primary CD8 T cell survival.

## Materials and Methods

### Mice

OT-I and OT-I × *OX40*-deficient (KO) TCR-transgenic mice, expressing a TCR composed of variable (Vβ5 and Vα2) chains responsive to an ovalbumin (OVA) 257–264 (SIINFEKL) epitope, were bred on a C57BL/6 background. C57BL/6 mice were purchased from Jackson Laboratory. This study was carried out in strict accordance with the guidelines of the Association for the Assessment and Accreditation of Laboratory Animal Care. The protocol was approved by the Institutional Animal Care and Use Committee (IACUC) of the Pennsylvania State University College of Medicine (Permit Number: 2007–127).

### Peptides and Antibodies

OVA 257–264 peptide was purchased from American Peptide Company (Sunnyvale, CA). Anti-OX40 (OX86) was produced from a hybridoma cell line obtained from the European cell culture collection (Wiltshire, UK). Mouse IL-2 and IFN-γ ELISA Kits were purchased from Biolegend (San Diego, CA). Anti-human/mouse A1 for Western blot (#7056) was purchased from Cell Signaling Technology (Beverly, MA). Anti-human/mouse Actin (C2, sc-8432) for Western blot was purchased from Santa Cruz Biotech.

### T Cells and APCs

Naive CD8^+^ T cells were purified from spleen and lymph nodes by using the murine Naive CD8a^+^ T Cell Isolation Kit (#130-096-543, Miltenyi Biotech, CA). The isolated cells were >90% CD8^+^ and >95% of these cells expressed the appropriate TCR and were also naive in phenotype. APCs were from spleens of syngeneic non-transgenic mice, by depleting T cells. APCs were treated with mitomycin c (100 µg/ml) for 30 min at 37°C.

### T Cell Cultures

Cultures were in 48-well plates containing 1 ml RPMI 1640 (Invitrogen) with 10% fetal calf serum (Omega Scientific, CA) [29]. Naive CD8^+^ T cells were plated at a density of 5×10^5^/ml with 2×10^6^/ml APCs in the presence of various concentrations of antigen. For determining the secondary responses, on day 5 of primary stimulation, 5×10^5^ T cells were isolated and recultured with 2×10^6^ APCs per ml. For Western blot, live CD8^+^ T cells were isolated from culture with CD8α (Ly-2) MicroBeads by Miltenyi Biotec (#130-049-401).

### Retroviral Transduction

MSCV-IRES-GFP/CA-IKKβ was generated previously [21]. MSCV-IRES-GFP/Flag-A1 was kindly provided by Dr. Ricky W. Johnstone [30]. Retroviral transduction was performed as described before [31]. 5×10^5^ T cells were stimulated with Ag/APCs. After 2 days, the supernatant was replaced with 1 ml viral supernatant containing 5 µg/ml Polybrene (Sigma), and the cells were spun for 1 hour at 32°C and incubated at 32°C for 8 hr. This was repeated the following day. Viral supernatant was removed and replaced with fresh medium, and T cells were re-cultured. Expression of GFP was determined by flow cytometry gating on Vβ5^+^ T cells. GFP-expressing T cells were purified by cell sorting using a FACSAria SORP high-speed cell sorter (BD Immunocytometry Systems, San Jose, CA).

### Adoptive Transfer and Tumor Challenge

T cells were cultured with Ag/APCs and transduced on day 2/3 with retroviral vectors [31]. Cells were recultured for 2 more days. GFP^+^ CD8^+^ T cells were sorted and 3×10^6^ sorted cells were injected *i.v.* into naive C57BL/6 mice. The following day, mice were challenged *s.c.* with 4×10^6^ B16-OVA tumor cells in PBS, or PBS without tumor cells as a control. Numbers of T cells were calculated based on total cell numbers in the spleen, draining lymph nodes (LN; inguinal, mesenteric, and paraaortic), and the peritoneal cavity, together with percentages of GFP^+^Vβ5^+^ cells visualized by using flow cytometry [29].

### Cytokine Secretion and Cell Recovery

Cytokines were measured by ELISA. T cell survival *in vitro* was determined by trypan blue exclusion [29].

### Immunoblotting

Live CD8^+^ cells were recovered by Ficoll treatment and positive selection with anti-CD8 microbeads (Miltenyi Biotec Inc). Cells were lysed in ice-cold RIPA Lysis Buffer (20 mM Tris-HCl (pH 7.5), 150 mM NaCl, 1 mM Na_2_EDTA, 1 mM EGTA, 1% Triton, 2.5 mM sodium pyrophosphate, 1 mM beta-glycerophosphate, 1 mM Na_3_VO4, and 1 µg/ml leupeptin) for 30 min. Insoluble material was removed and lysates used for Western blotting. Protein content was determined by Bio-Rad protein assay kit (Bio-Rad, Hercules, CA). Equal amounts (30 µg) were loaded onto 4–12% NuPage Bis-Tris precasting gels (SDS-PAGE), transferred onto PVDF membrane (Invitrogen), and immunoblotted. All blots were developed with the ECL immunodetection system (Amersham Pharmacia Biotech, Piscataway, NJ).

### Statistics

Unpaired *t* test or log rank test was used for the statistical analysis between groups and significance was set at 5%. All statistics were calculated using GraphPad Prism (San Diego, CA).

## Results

### Defective A1 Expression Correlates with Defective Survival of OX40 KO CD8 T Cells

**Figure 1:**
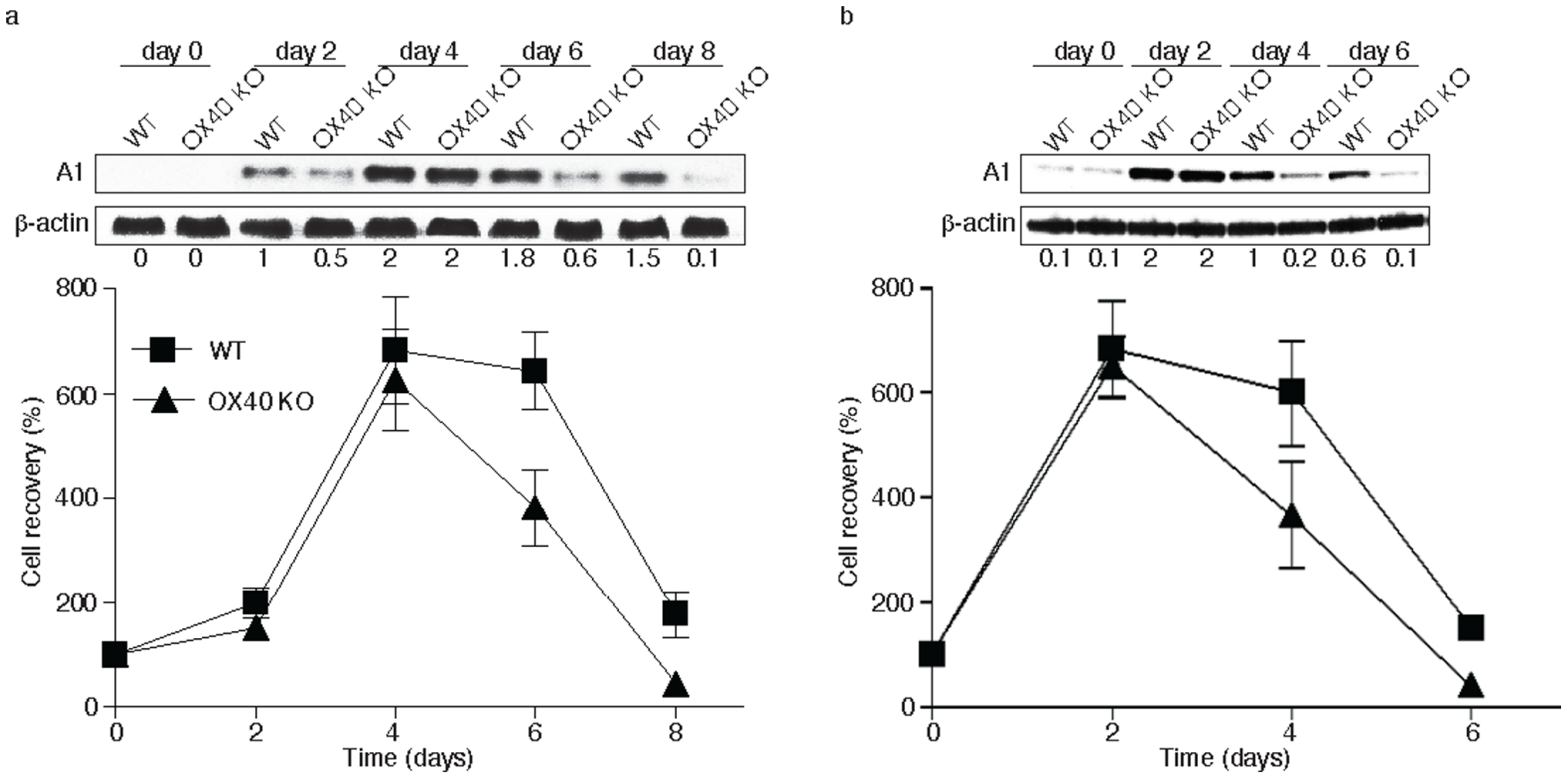
Defective A1 expression in OX40 KO CD8 T cells. (a) Naive CD8 T cells from wild-type (WT) or OX40-deficient (OX40 KO) OT-I TCR transgenic mice were stimulated *in vitro* with T-depleted APCs and OVA peptide over time. (b) After 6 days of primary culture, WT or OX40 KO T cells were restimulated with T-depleted APCs and OVA peptide for another 6 days. Data show the percentage T cell recovery, calculated based on assigning the input number of cells in each culture as 100%. Data are mean ± s.d from three experiments (*p*>0.05). Cell lysates were analyzed by immunoblotting for A1 and β-actin (top). Protein amounts were determined by densitometry and are shown relative to the expression in wild-type T cells transduced with control vector on day 2 or 4 (taken as 1).

OX40 is not constitutively expressed on naive CD8^+^ T cells, but up-regulated after 24 to 72 hours following activation; its ligand, OX40L, is also not expressed on resting antigen presenting cells, but is following their activation. OX40 KO CD8 T cells are sensitive to apoptosis and defective in their ability to proliferate during the initial primary response [2]. Our previous data have additionally shown that the defective A1 expression in OX40 KO CD4 T cells correlated with diminished survival [20]. To investigate the role of A1 in CD8 T cell survival driven by OX40, we analyzed A1 expression and the persistence of CD8 T cells from WT and OX40 KO TCR transgenic mice over several days *in vitro*. Primary CD8 T cell expansion and survival was similar between WT and OX40 KO T cells initially but cell recovery was markedly higher in WT T cells from day 6 to 8 (*p*>0.05). Importantly, this directly correlated with A1 expression that was not maintained in OX40 KO T cells at levels similar to WT T cells when assessed on days 6 and 8 (Fig. 1a). In addition, when the T cells were stimulated again in a secondary response, OX40 KO CD8 T cells expanded normally but were not maintained at WT numbers also correlating with defective A1 expression (Fig. 1b). These data suggest that A1 may be targeted by OX40 and regulates CD8 T cell survival.

### OX40 Signaling Up Regulates A1 Expression

**Figure 2:**
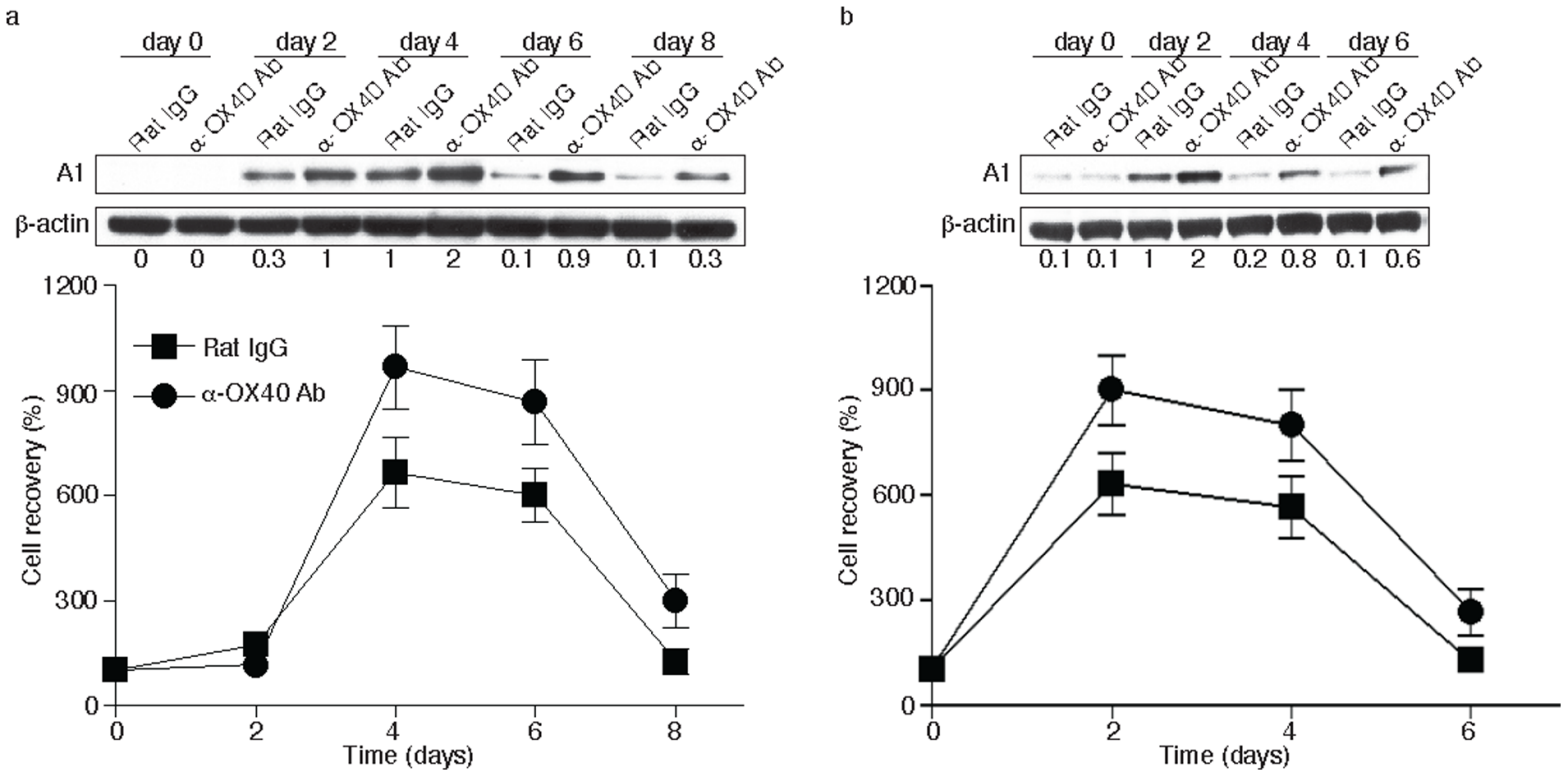
OX40 signaling sustains A1 expression and survival in CD8 T cells. (a) Naive CD8 T cells from WT OT-I TCR transgenic mice were stimulated *in vitro* with T-depleted APCs and OVA peptide in the presence or absence of 10 µg/ml agonist anti-OX40 or rat IgG added on day 0 and day 2. (b) After 5 days of primary culture, live T cells were isolated and restimulated with T-depleted APCs and OVA peptide in the presence or absence of 10 µg/ml agonist anti-OX40 or rat IgG added. Data show the percentage T cell recovery, calculated based on assigning the input number of cells in each culture as 100%. Data are mean ± s.d from three experiments (*p*>0.05). Cell lysates were analyzed by immunoblotting for A1 and β-actin (top). Protein amounts were determined by densitometry and are shown relative to the expression in wild-type T cells treated with rat IgG on day 4 or 2 (taken as 1).

To further explore whether OX40 signaling can regulate A1 expression in CD8 T cells, WT OT-I cells were stimulated with OVA peptide and APCs and cultures were supplemented with an agonist anti-OX40 antibody or rat IgG control on days 0, 1 and 2. Cell recovery was markedly higher in the anti-OX40 cultured group over time and again this correlated with enhanced A1 expression (Fig. 2a, *p*>0.05). Similarly, adding anti-OX40 into secondary cultures also enhanced the accumulation of CD8 T cells over time and correspondingly A1 expression was also maintained at elevated levels (Fig. 2b). These observations clearly indicate that A1 expression can be regulated by OX40 signaling and correlates with the propensity of CD8 T cells to accumulate or survive.

### A1 Restored Defective Survival of OX40 KO CD8 T Cells *in vitro*

**Figure 3:**
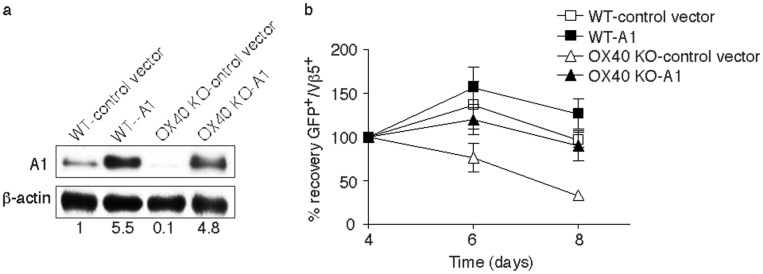
Retroviral transduction of OX40 KO CD8 T cells with A1 reverses defective survival *in vitro*. Naive CD8 T cells from WT or OX40 KO OT-I TCR transgenic mice were stimulated with T-depleted APCs and OVA peptide, and transduced on days 2/3 with retroviral vectors expressing GFP, or GFP with A1, and then recultured without any further stimulation. (a) On day 5 of primary culture, GFP^+^ T cells were sorted and analyzed for A1 and β-actin expression. Protein amounts were determined by densitometry and are shown relative to the expression in wild-type T cells transduced with control vector (taken as 1). (b) T cells recovered from primary cultures on day 5 were recultured in the absence of any further stimulation for 4 days. GFP^+^Vβ5^+^ T cell recovery normalized to take into account differences in initial transduction efficiency between cultures. Numbers of GFP^+^ cells present on day 4 were assigned a value of 100%, and numbers surviving on day 6 and day 8 were used to calculate the percentage recovery relative to day 4. Data represent the mean ± s.d. from three separate experiments (*p*>0.05).

To directly determine the relationship between A1 expression and OX40-mediated CD8 T cell survival, we retrovirally transduced antigen-stimulated T cells with an MSCV-GFP-IRES vector containing the full-length murine A1 gene [30]. After transduction, T cells were passively recultured in the absence of further antigen stimulation. A1 expression was dramatically lower in OX40 KO T cells than in WT cells at the start of this secondary culture coinciding with a greater loss of these CD8 T cells over 4 days (Fig. 3). Retroviral transduction enhanced the expression of A1 to equivalent levels in both WT and OX40 KO CD8 T cells. In line with this, monitoring the recovery of live GFP^+^ CD8 T cells showed that expression of A1 allowed OX40 KO T cells to persist similarly to WT T cells. Interestingly, WT CD8 T cells transduced with the A1 construct did not expand or survive better than WT T cells transduced with a control vector, even though the levels of A1 were approximately 5-fold increased. This suggests a threshold amount of A1 is required for CD8 T cell persistence, and also that the functional results in OX40 KO CD8 T cells were not simply an artifact of overexpression of A1 These observations strongly suggest that OX40-induced A1 expression can mediate CD8 T cell survival.

**Figure 4:**
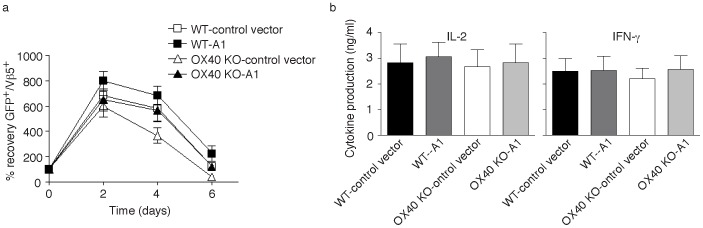
Forced expression of A1 restores the survival of OX40 KO CD8 T cells in secondary responses *in vitro*. Naïve CD8 cells from WT or OX40 KO OT-I TCR transgenic mice were stimulated with peptide and APCs. On day 2/3, T cells were transduced with retroviral vectors expressing GFP, or GFP with A1. On day 5 of primary culture, GFP^+^ CD8 T cells were sorted, and restimulated with APCs and peptide. (a) Recall survival, based on recovery of GFP^+^Vβ5^+^ T cells over time. Cell numbers present on day 0 were assigned a value of 100%, and cell numbers surviving on day 2, day 4 and day 6 were used to calculate the percentage recovery. Data represent the mean ± s.d. percentage changes from three separate experiments (*p*>0.05). (b) Recall IL-2 and IFN-γ production by ELISA at 40 h. Data are means ± s.d. from three experiments (*p*>0.05).

We further investigated whether forced expression of A1 could also reverse the defective recall responses of OX40 KO CD8 T cells in vitro. Effector CD8 T cells expressing A1 from primary naive cultures were therefore sorted based on GFP expression, and equal numbers were restimulated with antigen. OX40 KO CD8 T cells transduced with A1 displayed enhanced cell survival that essentially reversed the defective recovery that was observed over time compared to WT CD8 T cells (Fig. 4a). A1 did not promote greater IL-2 or IFN-γ production in OX40 KO T cells when restimulated with antigen (Fig. 4b), suggesting that the enhanced recall accumulation of OX40 KO CD8 T cells was not indirect through enhancing cytokine secretion.

### Active IKKβ Restores Defective A1 Expression and survival of OX40 KO CD8 T Cells *in vitro*

**Figure 5:**
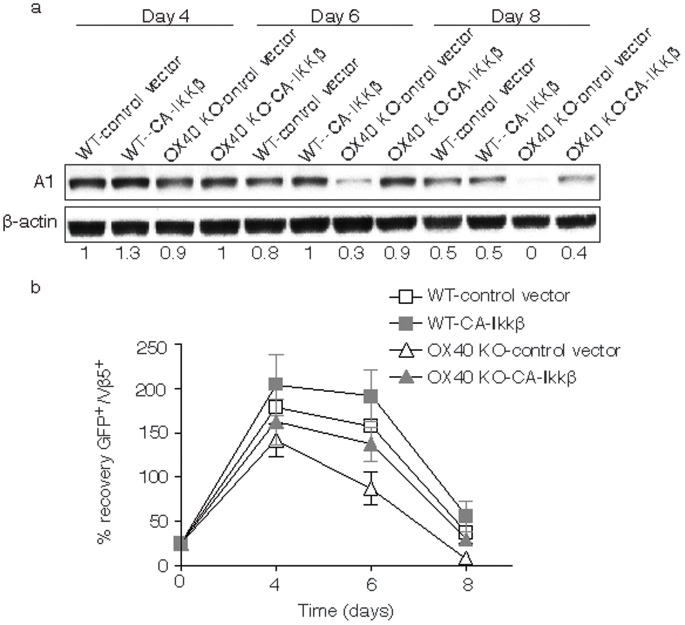
Active IKKβ regulates A1 expression in OX40 KO CD8 T cells. Naive CD8 cells from WT or OX40 KO OT-I TCR transgenic mice were stimulated with peptide and APCs. On day 2/3, T cells were transduced with retroviral vectors expressing GFP, or GFP with CA-IKKβ. On day 5 of primary culture, GFP^+^ CD8 T cells were sorted and restimulated with APCs and peptide. (a) On various days, GFP^+^ T cells were isolated and analyzed for A1 and β-actin. Protein amounts were determined by densitometry and are shown relative to the expression in wild-type T cells transduced with control vector (taken as 1). (b) Recall survival, based on recovery of GFP^+^Vβ5^+^ T cells over time. Cell numbers present on day 0 were assigned a value of 100%, and cell numbers surviving on days 4, 6 and 8 were used to calculate the percentage recovery. Data represent the mean ± s.d. percentage changes from three separate experiments (*p*>0.05).

Our previous data showed that activation of NF-κB by OX40 contributed to antigen-driven CD4 T cell expansion and survival [21]. To determine whether activation of NF-κB regulated A1 expression, we retrovirally transduced WT and OX40 KO CD8 T cells with an active version of IKKβ. IKKβ-GFP-expressing T cells were sorted and restimulated with antigen, and then CD8 cell lysates were examined by Western blot. OX40 KO CD8 T cells transduced with control vector had dramatically reduced amounts of A1 at late times, which were essentially restored back to WT T cell levels by expressing active IKKβ (Fig. 5a). Furthermore, active IKKβ also restored defective survival of OX40 KO CD8 T cells (Fig. 5b). Thus, activation of NF-κB by OX40 may control A1 expression, which contributes to the antigen-driven survival of CD8 T cells.

### A1 Reverses Defective Survival of OX40 KO CD8 T Cells *in vivo* and Restores the Ability of OX40 KO CD8 T Cells to Suppress Tumor Growth

**Figure 6:**
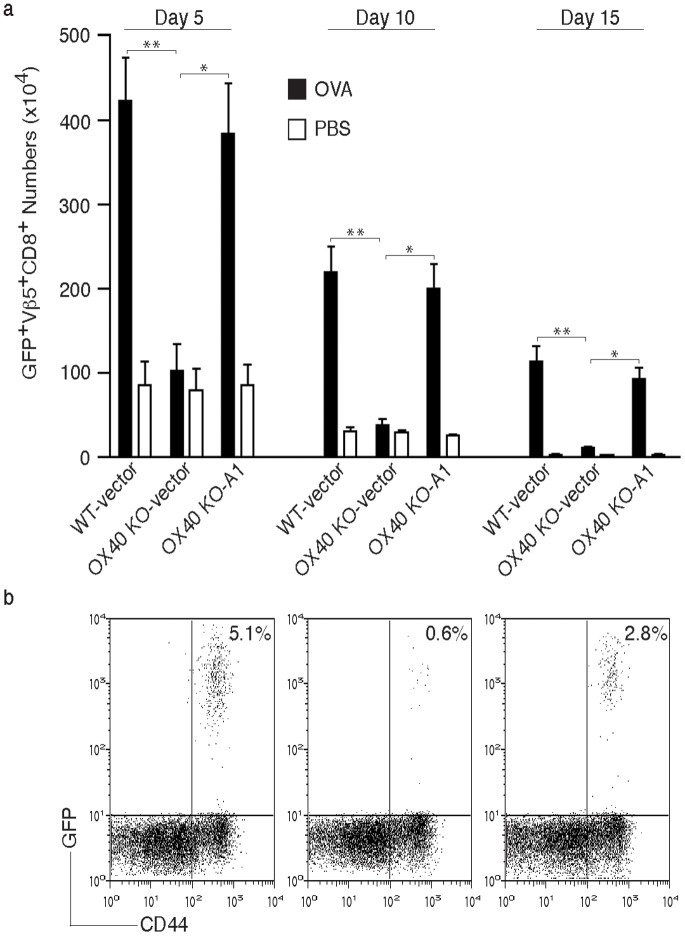
Forced expression of A1 restores the ability of OX40 KO CD8 T cells to accumulate and survive over time *in vivo*. Naive CD8 T cells from WT or OX40 KO OT-I TCR transgenic mice were stimulated with APCs/peptide. On day 2/3, T cells were transduced with retroviral vectors expressing GFP, or GFP with A1. On day 5 of primary culture, GFP^+^ CD8 T cells were sorted and adoptively transferred into naive C57BL/6 recipient mice that were subsequently challenged *i.p.* with whole OVA protein in PBS (filled bars) or PBS alone (open bars). (a) On days 5, 10, and 15, GFP^+^Vβ5^+^CD8^+^ T cells were enumerated from pooled lymph nodes and spleen. Data are mean number of GFP^+^Vβ5^+^CD8^+^ cells ± s.d from four individual mice and representative of three experiments (* *P*<0.05, ** *P*<0.01, Student's unpaired *t*-test). (b) At day 15, percentage of GFP^+^CD44^+^ T cells was analyzed by flow cytometry, after gating on live CD8^+^ T cells in the spleen. Results are representative of three experiments.

To show that A1 can control CD8 T cell accumulation and survival in a truly physiological setting, WT and OX40 KO A1 gene-transduced OT-I effector CD8 T cells were adoptively transferred into syngeneic recipients. These mice were subsequently challenged with OVA protein. OX40 KO CD8 T cells expanded and survived markedly less than WT T cells over 15 days in lymph nodes or spleen, and this defect was rescued by forced expression of A1 (Fig. 6) supporting the *in vitro* results.

**Figure 7:**
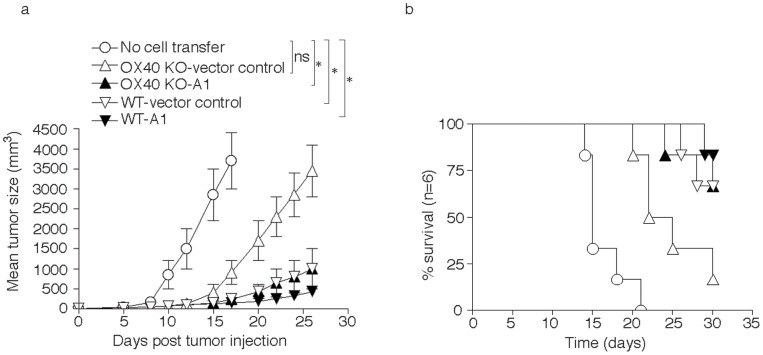
Expression of A1 restores the ability of OX40 KO CD8 T cells to suppress tumor growth *in vivo*. Naive CD8^+^ T cells from WT or OX40 KO OT-I TCR transgenic mice were stimulated with APCs/peptide. On day 2/3, T cells were transduced with retroviral vectors expressing GFP, or GFP with A1. On day 5 of primary culture, GFP^+^ CD8^+^ T cells were sorted and adoptively transferred into naive recipient mice that were subsequently injected with B16-OVA melanoma cells *s.c.* in the flank region. (a) Tumor growth was monitored over time. Tumor volume was calculated as follows: V = length×width^2^×0.52. Data are mean tumor size ± s.d from six individual mice and representative of three experiments (* *P*<0.05, Student's unpaired *t*-test). (b) Mouse survival was assessed over 30 days. Kaplan–Meier survival analysis indicated significantly increased survival in mice (*n* = 6) receiving OX40 KO CD8^+^ T cells transduced with A1 compared to mice receiving OX40 KO CD8 T cells with vector control (logrank test, *P*<0.05).

To extend this to a clinically relevant setting, A1-transduced (GFP-sorted) WT or OX40 KO CD8 T cells were adoptively transferred into syngeneic recipients that were subsequently injected *s.c.* with B16 tumor cells expressing OVA. WT T cells transduced with control vector delayed tumor growth and prolonged mouse survival equivalently to WT T cells transduced with A1. OX40 KO CD8 T cells bearing the control vector were only modestly effective at suppressing tumor growth, correlating with their reduced survival, whereas the A1 gene-transduced OX40 KO CD8 T cells displayed a similar activity to WT T cells (*P*<0.05, Student's unpaired *t*-test, Fig. 7a). In addition, significantly more mice survived up to 30 days after the injection of B16-OVA tumor cells that received the A1-gene transduced OX40 KO CD8 T cells compared to control vector-transduced OX40 KO CD8 T cells (16.7% *vs* 66.7%; *P*<0.05, log rank test), and survival was similar to mice receiving the vector-transduced WT CD8 T cells (Fig. 7b). These data strongly support the conclusion that defective CD8 T cell persistence that is observed as a result of a lack of OX40 signals is at least in part explained by a reduction in expression of the pro-survival Bcl-2 family molecule A1.

## Discussion

The A1 protein is thought to be involved in a variety of cellular activities such as embryonic development, homeostasis, and tumorigenesis [32]. It has been described to control the release of cytochrome c from mitochondria and block caspase activation by binding to and antagonizing the activity of pro-apoptotic members of the Bcl-2 family, including Bid and Bim. The A1 gene is also a direct transcriptional target of NF-κB and has been reported to be induced in response to a variety of inflammatory mediators and extracellular signals, such as granulocyte-macrophage colony-stimulating factor (GM-CSF), CD40, phorbol ester, LPS, and inflammatory cytokines like TNF and IL-1β. In this paper, we now show that OX40 signals in CD8 T cells promote expression of A1, and A1 can play a critical role in the persistence and survival of CD8 T cells responding to antigen.

The physiologic relevance of A1 in lymphocyte homeostasis is poorly understood. In mice, the A1 gene locus has undergone quadruplication that has resulted in the production of three functional A1 genes encoding A1-a, A1-b, and A1-d isoforms, and one pseudogene, A1-c. Among the three murine A1 genes, the exon sequences display 96% sequence identity at the nucleotide level and 97% identity at the protein level. A1-c only encodes a truncated version of the A1 protein that contains a BH1-domain. Interestingly, the different isoforms of A1 present different patterns of expression in the hematopoietic compartment, which has complicated studies of multiple lineages of cells with A1 knockout mice [22]. A1-a is poorly expressed in both T and B lymphocytes [33] compared to the other A1 isoforms. A deficiency of A1-a in mice leads to augmented apoptosis of mast cells after allergen-driven activation and a diminished acute inflammatory response *in vivo* [34], [35], but the significance of A1-a versus the other A1 isoforms to lymphocyte responses is not clear. In contrast to mice, the human genome only has one A1/Bfl-1 gene, whose expression has been correlated to the pathology of lymphomagenesis [36]. In conclusion, the role of A1 in lymphocyte activities is largely unknown, particularly in relation to other similar members of the Bcl-2 family such as Bcl-2 and Bcl-xL.

We previously reported that the PI3K/PKB and NF-κB pathways are the main target of OX40 signals that regulate the proliferation, expansion and long-term survival of CD4 T cells [20], [31], [37]–[39]. Through activating these pathways, OX40 controls CD4 T cell expansion and proliferation by promoting survivin and aurora B kinase expression [31], [40], and sustains T cell survival in part by regulating the expression of Bcl-2 and Bcl-xL [37]. However, whether OX40 signals regulate the same pathways and intracellular targets in CD8 T cells has not been investigated thoroughly. OX40-deficient effector CD8 T cells were previously found to be impaired in expressing Bcl-xL, and forced expression of Bcl-xL into OX40 KO or WT CD8 T cells augmented their survival and anti-tumor activity [3], [29]. Our current study identified that A1, which was regulated by active IKKβ in primary CD8 T cells, is also an important intracellular target of OX40 signals that can promote CD8 T cell survival. We also found A1 expression was defective in OX40-deficient CD4 T cells but did not investigate any contribution of A1 to the CD4 T cell response [20], [21]. These results suggest that OX40 signals may use the PI3K/PKB and NF-κB pathways to regulate both CD4 and CD8 T cell activities. However, whether OX40 signals also promote CD8 T cell proliferation via survivin or aurora B kinase needs to be addressed in the future.

Our finding that A1 is regulated by OX40 in primary CD8 T cells is in line with prior data showing that A1 expression was also induced by signaling through 4-1BB (CD137), another TNFR family member that promotes CD8 T cell survival [41]. 4-1BB ligation also increased the expression of Bcl-xL, suggesting that both OX40 and 4-1BB may control the expression of the same Bcl-2 family members to promote long-term T cell survival. Out of the three primary anti-apoptotic proteins (Bcl-xL, Bcl-2, and A1), their respective contributions to T cell survival in any given immune response are however unclear. We found that *in vitro* in both CD4 and CD8 T cells, all three were not maintained in the absence of OX40 signals, or up-regulated in the presence of OX40 signaling triggered with an agonist anti-OX40 antibody. These observations imply that the each contributes an important function and the quantitative expression of all three combined will dictate the ability of T cells to persist. This notion is supported by the finding that forced expression of A1, Bcl-xL, or Bcl-2 can almost fully restore the survival of OX40-deficient T cells. Conversely, the knockdown of the each in wild type T cells with shRNA does not significantly impact the effect on OX40 signaling in cell survival in the model systems we have employed. However, it is possible that these molecules also can display redundant functions in lymphocytes and their relative importance will be a product of the signaling receptors like OX40 that can promote their expression and other inflammatory factors that might favor expression of one molecule over another as T cells respond *in vivo*. A comparative study using gene transduction of OX40 KO CD8 T cells with varying levels of one, two, or three of these genes may address the overlapping or redundant functional role of these proteins that mediate CD8 T cell survival.

In conclusion, our current study provides evidence that OX40 signaling can promote long-term primary CD8 T cell survival at least in part by regulating A1 expression. Moreover, this study also provides data that IKKβ/NF-κB activation may mediate OX40-driven A1 expression in CD8 T cells. As agonist antibodies to OX40 are currently in clinical trials for cancer, these data have implications for understanding how OX40 initiates signaling events in CD8 T cells that might determine their therapeutic efficacy.
